# Variations and patterns in germline and somatic testing by age in ovarian, primary peritoneal, and fallopian tube cancer patients: a descriptive analysis in a large, real-world clinical and genetic data registry

**DOI:** 10.1016/j.gore.2026.102091

**Published:** 2026-05-02

**Authors:** Martins Ayoola, Alexandra Smick, Miles Morrow, Chinmayi Aryasomayajula, Caitlin Johnson, Daniel Kapp, John K. Chan, Dana M. Chase

**Affiliations:** a Department of Obstetrics and Gynecology, University of California, Los Angeles, Los Angeles, CA, USA; b University of California, Santa Cruz, Santa Cruz, CA, USA; c Kaiser Permanente Santa Clara, Santa Clara, CA, USA; d California Pacific Medical Center Research Institute, Sutter Health, San Francisco, CA, USA; e Stanford University, Palo Alto, CA, USA

**Keywords:** HRD testing and age, Intermediate GIS score and age

## Abstract

**Objective:**

To evaluate the relationship between genomic instability score and age distribution in ovarian, primary peritoneal, and fallopian tube cancers and to characterize age-related patterns in germline, homologous recombination deficiency (HRD), and somatic tumor testing using a large real-world clinical registry.

**Methods:**

Patients with ovarian, primary peritoneal, and fallopian tube cancers who underwent germline and/or somatic testing were identified from the Myriad Collaborative Research Registry (MCRR), version 4 (March 2025). Genomic instability scores (GIS) and tumor mutation profiles were analyzed across four age groups (<50, 50–59, 60–69, and ≥70 years). HRD positivity was defined as GIS ≥ 42. Testing patterns were evaluated by ordering specialty. Comparisons were performed using chi-square testing with significance defined as p < 0.05.

**Results:**

Among 110,119 patients with ovarian, peritoneal, or fallopian tube cancer, HRD positivity decreased with increasing age (36.2% in <50 years, 36.4% in 50–59 years, 30.9% in 60–69 years, and 24.2% in ≥70 years; p < 0.0001). Median GIS was similar across age groups but demonstrated narrower distribution ranges in older patients. Subgroup analysis of patients with intermediate GIS scores (35–41) showed increasing prevalence with advancing age. Gynecologic oncologists ordered significantly more HRD tests than medical oncologists; however, HRD positivity rates were similar between specialties. Peritoneal cancer diagnoses increased with age.

**Conclusion:**

Younger patients demonstrated higher rates of HRD positivity compared to older patients despite similar median GIS distributions. These findings may have implications for biomarker-driven therapeutic selection and clinical trial generalizability, particularly in older ovarian cancer populations. Further investigation is needed to understand age-related molecular differences and optimize testing and treatment strategies across age groups.

## Highlights

• Homologous recombination deficiency (HRD) positivity decreases with increasing age.
• Intermediate genomic instability scores increase in frequency with advancing age.
• HRD testing is ordered more frequently by gynecologic oncologists with similar positivity rates with medical oncologists.
• Age-related molecular differences may influence PARP inhibitor use and clinical trial applicability.

## 1 Background

Ovarian cancer remains a leading cause of gynecologic cancer-related mortality with a 5-year survival rate of approximately 50%. While often diagnosed at an advanced stage, there has been a declining trend in both incidence and death rates, with new cases at a rate of 10.3 per 100,000 women per year and death rates at 6.3 per 100,000 women per year (Cancer Stat Facts: Ovarian Cancer). Several factors contribute to this positive trend, including development of more effective therapeutics and opportunistic risk reducing procedures.

The introduction of poly-ADP ribose inhibitors (PARPi) has demonstrated a significant decrease in risk of disease progression or death, particularly in patients with germline *BRCA* mutations, with a remarkable 70% reduction (Moore et al., 2018). Germline BRCA1 and BRCA2 mutations represent the most well-characterized contributors to homologous recombination repair (HRR) deficiency and are present in approximately 15–22% of patients, with somatic mutations account for an additional 5–7% (Witjes et al., 2022, Andrikopoulou et al., 2022). Beyond BRCA1/2, alterations in other HRR related genes, including RAD51C/D, BRIP1, PALB2, and CHEK2 account for the remaining homologous repair defect (HRD) cases. However, these are less consistently used as predictive biomarkers for PARPi response.

Given the limitations of single-gene testing, companion diagnostic tests have been developed to identify patients who may still benefit from PARPi especially when germline testing is negative. These tests focus on incorporating measures of genomic instability, including loss of heterozygosity (LOH), telomeric allelic imbalance (TAI), and large-scale state transitions (LST) to generate a composite genomic instability score (GIS). Numerous studies have shown significant improvement in progression-free and overall survival benefits in patients with HRD, whether in the front line or recurrent setting (González-Martín et al., 2019, Ray-Coquard et al., 2019). A GIS threshold of ≥42 has been established as positive for high genomic instability and demonstrated potential benefit in patients who used PARP inhibitors, even in the absence of BRCA mutation.

To date, no study has explored the connection between genomic instability/HRD expression and age distribution. This is important as it has been shown that younger ovarian cancer patients (≤65 years old) tend to survive longer than the older patients. In addition, a noticeable trend in the PARPi trials is the higher enrollment of younger patients, making the results more applicable to patients ≤65 years old. Consequently, these trials may not fully reflect HRD results in the older population or the potential benefits of PARPi.

Using a large real-world clinical and genetic registry dataset, we sought to evaluate the association between genomic instability and age distribution among patients with ovarian, primary peritoneal, and fallopian tube cancers. Additionally, we examined age-related patterns in germline, somatic, and HRD testing utilization, including differences based on ordering provider specialty and geographic distribution.

## 2 Methods

### 2.1 The Myriad Collaborative Research Registry (MCRR)

The MCRR is a unique, web- based, IRB-approved registry of real-world clinical testing results that contains de-identified clinical and molecular testing results for over 1.2 million patients with a broad list of cancer diagnoses. The MCRR can also provide data about the frequency and outputs from national test usage for patient care, leading to possible analyses of patterns of care.

Clinical and molecular data are collected at the time of test ordering through standardized requisition forms completed by the ordering provider. These data include patient demographics, self-reported ancestry/ethnicity, cancer diagnosis, and molecular testing results. Diagnoses are assigned by the ordering provider and are not independently adjudicated within the registry, which may introduce potential misclassification. Molecular data available within the MCRR include germline testing (myRisk® Hereditary Cancer Test), homologous recombination deficiency (HRD) assessment (myChoice® CDx), and somatic tumor profiling (Precise Tumor™).

The myRisk Herediatry Cancer test is a multigene panel that identifies germline variants associated with 11 known hereditary cancers. Germline variants are categorized as suspected deleterious, deleterious, variants of unknown significance, or low penetrance.

The MyChoice CDx HRD test uses a tumor genomic instability score (GIS) based on loss of heterzygosity (LOH), telomeric allelic imbalance (TAI), and large-scale state transitions (LST) to provide a comprehensive assessment of HRD status.

The Precise Tumor test uses comprehensive genomic profiling to identify somatic variants by analyzing the DNA of tumors. Tumor variant classifications are reported based on joint consensus recommendations from the Association for Molecular Pathology, the American Society of Clinical Oncology, and the College of American Pathologists. Classifications are defined as: IA (FDA approved therapy or practice guidelines for patient’s tumor type); IB (Consensus in the field based on well powered studies for patient’s tumor type); IIC (FDA approved therapy or practice guideline in other tumor type(s), evidence from multiple small published studies, or based on availability of investigational therapies); and IID (case reports or preclinical studies).

### 2.2 Study population and data extraction

Data was extracted from the MCRR version 4 (March 2025), starting with general registry information, such as description of cancer types (spanning 2013–2025). Descriptive data was then extracted for patients with the top of gynecologic cancers, including for patients who received germline (years 2013–2025), HRD status (2017–2025), and/or somatic (years 2022–2025) testing.

Patients were stratified into four predefined age groups: <50, 50–59, 60–69, and ≥70 years. Cancer diagnoses were evaluated both individually and in grouped analyses, with ovarian and fallopian tube cancers combined in select analyses to account for potential misclassification between these sites.

Genomic instability scores (GIS) derived from the myChoice® CDx assay were analyzed across age groups. HRD positivity was defined as a GIS ≥ 42, consistent with validated thresholds. A subgroup analysis was performed among patients with intermediate GIS values (35–41) to further evaluate age-related distribution patterns.

Age-stratified analyses were also conducted for germline pathogenic variants and somatic tumor alterations across commonly implicated genes in homologous recombination repair and tumorigenesis pathways.

### 2.3 Outcomes

The primary outcome was the association between age and HRD status, as defined by GIS. Secondary outcomes included:

• Distribution of GIS values across age groups
• Prevalence of germline pathogenic variants by age
• Prevalence of somatic tumor variants by age
• Patterns of molecular testing utilization by provider specialty

### 2.4 Statistical analysis

Categorical variables were compared using the chi-square test. Age-stratified trends in mutation prevalence and HRD positivity were evaluated across the four predefined age groups. All statistical tests were two-sided, with a significance threshold of p < 0.05.

Analyses were conducted using aggregated registry data. Due to the de-identified and observational nature of the dataset, adjustment for potential confounders was limited to stratified analyses.

## 3 Results

### 3.1 Gynecologic cancers

The top five gynecologic cancer diagnoses reported in the MCRR include ovarian, endometrial/uterine, cervical, peritoneal, and fallopian tube. Of the 16,731 patients with one of these diagnoses and MyChoice CDx testing, 5179 had positive HRD status and 11,652 had negative HRD status. GIS scores ranged 0–104, with a median of 27.

Total patient numbers for these diagnoses are 104,968 for ovarian, 41,454 for endometrial/uterine, 12,269 for cervical, 5,604 for peritoneal, and 5,465 for fallopian tube. The number of germline tests available and age (age; interquartile range, IQR) per diagnosis was: 96,275 (57; 46–67) for ovarian, 40,812 (54; 43–63) for endometrial/uterine, 12,094 (35; 27–47) for cervical, 4422 (65; 57–72) for peritoneal, and 4034 (62; 53–70) for fallopian tube. Finally, the reported number of somatic tests and age (median; IQR) for each diagnosis was: 2020 (63; 67–71) for ovarian, 1257 (62; 42–70) for endometrial/uterine, 192 (50; 40–60) for cervical, 320 (66; 58–72) for peritoneal, and 330 (65; 57–71) for fallopian tube.

**Fig. 1:**
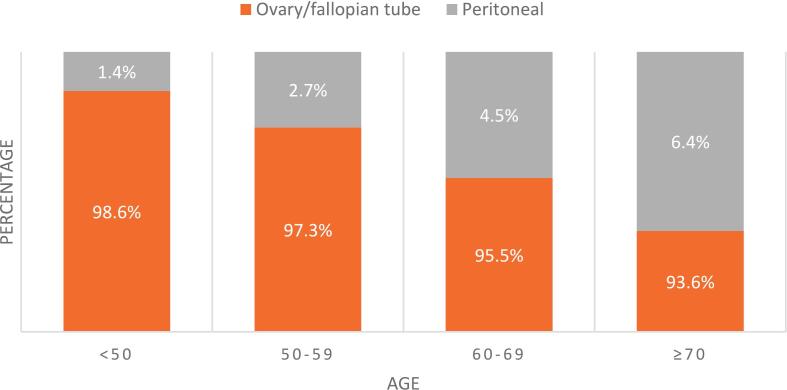
Cancer diagnosis by age.

There was an increase in peritoneal diagnosis with age <50, 50–59, 60–69, and ≥70 at 1.4%, 2.7%, 4.5%, and 6.4% (p < 0.0001) (Fig. 1, Table 1, and Table 2).

**Table 1:** Demonstrates the cancer site of diagnosis for Myriad testing by age.

Number of patients (N = 110,119)	Age				P-value
	<50	50–59	60–69	≥70	
	31,739 (%)	27,731 (%)	28,470 (%)	22,179 (%)	
Ovary/Fallopian tube	31,294 (98.6)	26,977 (97.3)	27,188 (95.5)	20,911 (93.6)	
Peritoneal	445 (1.4)	754 (2.7)	1282 (4.5)	1268 (6.4)	P < 0.00001

**Table 2:** Highlights the ancestral breakdown of key demographics.

Ancestry	Age			
	<50	50–59	60–69	≥70
	33,034 (%)	31,010 (%)	30,735 (%)	23,247 (%)
None specified	4,821 (14.6)	4,651 (15)	5,314 (17.3)	4,571 (19.7)
Western/Northern Europe	10,688 (32.4)	11,407 (36.8)	10,744 (34.9)	7,071 (30.4)
White	5,018 (15.2)	4,690 (15.1)	5,807 (18.9)	5,729 (24.6)
Hispanic	2,950 (8.9)	1,852 (5.9)	1,384 (4.5)	824 (3.5)
Eastern European	2,394 (7.3)	2,285 (7.4)	2,081 (6.8)	1,393 (5.9)
Black	1,643 (4.9)	1,410 (4.6)	1,388 (4.5)	834 (3.6)
Asian	1,364 (4.1)	926 (2.9)	653 (2.1)	406 (1.8)
Native American	1296 (3.9)	940 (3.0)	807 (2.6)	519 (2.2)

**Fig. 2:**
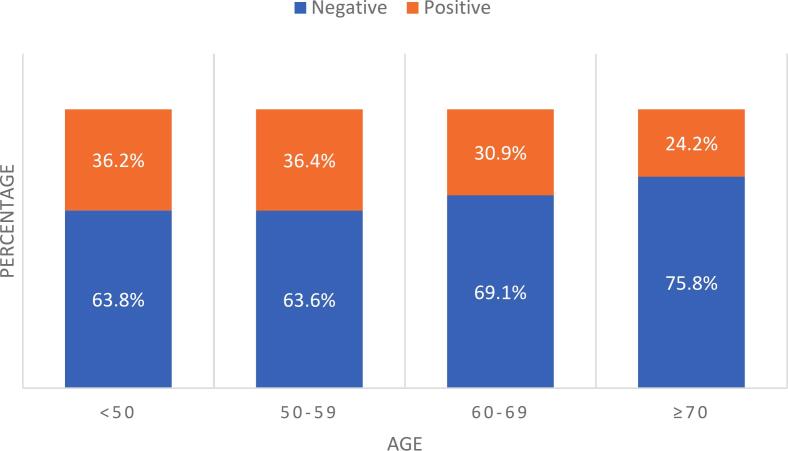
GIS by age.

Based on age, those who were <50, 50–59, 60–69, and ≥70 had rates of positive HRD at 36.2%, 36.4%, 30.9%, and 24.2% respectively (p < 0.0001). The median GIS for patients <60 was 27 [IQR = 10,56] whereas median score for patients ≥60 was 28 [IQR = 17,44] (Fig. 2).

When looking at a subgroup of patients with GIS of 35–41, we were able to identify that the number of patients within this category increased with each decade of life (p < 0.0001). We also noticed that the median score and IQR for the younger patients tend to be on the higher side whereas older patients generally had a slightly lower median and range (Table 3).

**Table 3:** Depicts characteristics of a subgroup of patients with gis between 35–41.

GIS 35–41	Age				P-value
	<50	50–59	60–69	≥70	
Number of patients	48	162	348	380	P < 0.0001
Median age (IQR)	47 (43,48)	57 (54,58)	64 (62,67)	75 (72,78)	
Ovary/Fallopian tube	47	170	372	383	
Peritoneum	5	13	34	47	
Median GIS (IQR)	39 (37,40)	38 (36,39)	38 (36,39)	38 (36,39)	
	**<60**	**≥60**			
Number of patients	210	728			
Median score	38	38			

When looking at germline gene variants, we noted the following: The prevalence of BRCA1 mutations decreased significantly with increasing age, whereas BRCA2 and BRIP1 mutations increased (p < 0.0001). Several moderate-risk genes, including ATM, RAD51C, RAD51D, and TP53, also demonstrated significant age-associated variation. CHEK2, STK 11, PTEN, and PALB2 did not demonstrate a significant trend (Table 4).

**Table 4:** Age-stratified distribution of pathogenic germline variants. Data represents pathogenic and likely pathogenic germline variants identified through multigene panel testing.

Germline genes	<50 (%)	50–59 (%)	60–69 (%)	≥70 (%)	P-value
BRCA1	28.9	24.1	22.1	8.4	<0.0001
BRCA2	10.9	17	23.5	15.6	<0.0001
BRIP1	2.7	3.6	4.5	6.2	<0.0001
CHEK2	3.3	3	2.8	3.6	>0.05
PALB2	1.2	1.1	1.5	1.7	>0.05
ATM	4	4.3	4.6	6	<0.01
RAD51C	0.9	1.6	2	1.6	<0.05
RAD51D	1.6	1.8	2	2.1	<0.05
TP53	1.2	1.3	1.4	2.4	<0.05
STK11	0.8	0.7	0.7	1.1	>0.05
PTEN	0.3	0.2	0.2	0.2	>0.05

Somatic tumor variants demonstrated distinct age-associated patterns. TP53 mutations increased significantly with age, whereas ARID1A and PTEN mutations decreased (all p < 0.001). Somatic BRCA1 mutations decreased while BRCA2 mutations increased across age groups (both p < 0.001). KRAS mutations showed modest variation (p = 0.016), whereas PIK3CA and RAD51c did not demonstrate a statistically significant across age groups (Table 5).

**Table 5:** Age-stratified distribution of pathogenic somatic variants.

Somatic genes	<50 (%)	50–59 (%)	60–69 (%)	≥70 (%)	P-value
BRCA1	27.6	24.3	22.2	16.6	<0.0001
BRCA2	8.3	13.3	16.7	16.3	<0.001
TP53	13.5	13.9	16.6	21.7	<0.0001
ARID1A	6	3.7	2.9	1.6	<0.0001
KRAS	4.7	2.9	2.6	2.5	0.016
PIK3CA	4.1	3.9	3	2.1	>0.05
PTEN	3.6	1.7	1.5	0.9	<0.001
RAD51C	0.2	0.1	0.1	0.1	>0.05

Molecular testing patterns were compared between the two subspecialty providers, gynecologic oncologists (GO) and medical oncologists (MO). Median age of these patients was 64 years old (IQR, 55, 72). GO ordered 3003 MyChoice (R) CDx HRD Diagnostic Test (73.5%) versus (vs) 1084 (26.5%) by medical oncologists (MO). GO ordered more tests per disease site than MO. However, when comparing percentage of test per cancer diagnosis, MO had a higher percentage of patients with ovarian and peritoneal cancer diagnoses. The GO tests had 30.2% HRD status vs 29.4% for MO tests (p = 0.65) (Table 6).

**Table 6:** Disease and HRD characteristics based on ordering physician.

	Gynecologic oncologist	Medical oncologist	P-value
**Cancer diagnosis**	3453 (%)	1230 (%)	
Ovary/Fallopian tube	3150 (91.2)	1111 (90.3)	P = 0.83
Peritoneal	303 (8.8)	119 (9.7)	P = 0.38

**HRD status**			P = 0.65
Positive	906 (30.2)	319 (29.4)	
Negative	2097 (69.8)	765 (70.6)	

## 4 Discussion

The MyChoice assay, found in the MCRR, is based on sequencing over 25,000 single nucleotide polymorphism (SNP) loci spread across the human genome and is highly reproducible (96.9% concordant) regardless of whether the assay is performed by the central Myriad laboratory or an academic institution following a technology transfer with a sensitivity of 94.6% and specificity of 98.4% (Denkert et al., 2022). This highlights the low false positive and negative rates of MyChoice GIS testing, which appear consistently across age groups, making it less likely to report a high false positive or negative result in older patients.

Consistent with prior reports, we observed higher HRD positivity among younger patients. Interestingly, this finding differs from the theoretical expectation that genomic instability may increase with aging. Several potential explanations may contribute to this observation. The increasing proportion of primary peritoneal carcinoma with age may contribute to variability in genomic profiles as it pertains to biopsy site, tissue quality, and sampling adequacy. Additionally, differences in treatment exposure, including the potential use of neoadjuvant chemotherapy, may alter tumor biology and influence genomic testing results. Differences between primary tumor and metastatic site sampling may also contribute, although this could not be evaluated within the available dataset.

These findings raise important clinical questions regarding the interpretation and utilization of HRD testing in older patients. Because BRCA-associated tumors are more frequently observed in younger populations and are associated with higher genomic instability, age-related differences in tumor biology may influence treatment selection and expected response to PARP inhibition. These findings are consistent with the higher prevalence of BRCA1 mutations in younger patients observed in our dataset, which are known to be associated with HRD positivity. Furthermore, older patients are underrepresented in many phase III clinical trials, as most patients enrolled are under 65 years old, limiting the generalizability of trial results to this population. Differences in pharmacokinetics, treatment tolerance, and comorbidity burden further complicate treatment decision-making in older patients. Fortunately, newer therapeutics, such as antibody-drug conjugates, are on the horizon, with potentially more manageable toxicities.

Our study also demonstrated differences in molecular testing patterns between gynecologic oncologists and medical oncologists. Although gynecologic oncologists ordered more HRD testing overall, diagnostic yield was similar between specialties. Notably, testing patterns shifted with age, with relatively fewer tests ordered by gynecologic oncologists and increased testing ordered by medical oncologists in older patients. These differences may reflect evolving referral patterns, differences in practice settings, or increased involvement of medical oncologists in the management of gynecologic oncology patients. The clinical drivers underlying these differences warrant further investigation.

The primary strength of this study is the use of a large, real-world clinical registry. However, several limitations must be acknowledged. As a registry-based analysis, detailed clinical variables were limited. Timing of tissue acquisition relative to diagnosis or recurrence was not available, nor was treatment sequencing, including use of neoadjuvant chemotherapy or primary cytoreduction. These factors may influence tumor biology and genomic testing results and should be explored in future prospective studies.

Overall, these findings are consistent with differences in BRCA mutation prevalence across age groups. These findings may have implications for biomarker-driven treatment selection and clinical trial design, particularly in older patient populations.

## 5 Conclusions

In this large real-world registry analysis, HRD positivity was more frequently observed in younger patients with ovarian, primary peritoneal, and fallopian tube cancers. While median genomic instability scores were similar across age groups, HRD-positive tumors were less common in older patients. These findings may have important implications for biomarker-driven therapeutic selection and highlight potential limitations in extrapolating clinical trial data to older patient populations. Further research is needed to better characterize age-related molecular differences and optimize treatment strategies across the full age spectrum of ovarian cancer patients.

## 6 Declaration of generative AI and AI-assisted technologies in the manuscript preparation process

During the preparation of this work the author used ChatGPT strictly for proofreading. After using this tool, the author reviewed and edited the content as needed and take full responsibility for the content of the published article.

## CRediT authorship contribution statement

**Martins Ayoola:** Writing – review & editing, Writing – original draft, Formal analysis, Data curation. **Alexandra Smick:** Writing – review & editing. **Miles Morrow:** Writing – review & editing. **Chinmayi Aryasomayajula:** Writing – review & editing. **Caitlin Johnson:** Writing – review & editing. **Daniel Kapp:** Writing – review & editing, Validation, Supervision. **John K. Chan:** Writing – review & editing, Validation, Supervision, Conceptualization. **Dana M. Chase:** .

## Declaration of competing interest

The authors declare that they have no known competing financial interests or personal relationships that could have appeared to influence the work reported in this paper.

## References

[b0005] Andrikopoulou A., Zografos E., Apostolidou K., Kyriazoglou A., Papatheodoridi A.M., Kaparelou M., Koutsoukos K., Liontos M., Dimopoulos M.A., Zagouri F. (2022). Germline and somatic variants in ovarian carcinoma: a next-generation sequencing (NGS) analysis. Front. Oncol..

[b0015] Cancer Stat Facts: Ovarian Cancer. National Cancer Institute. Surveillance, Epidemiology, and End Results Program.

[b0030] Denkert C., Romey M., Swedlund B., Hattesohl A., Teply-Szymanski J., Kommoss S., Kaiser K., Staebler A., du Bois A., Grass A., Knappmeyer C., Heitz F., Solimeno C., Ebel T., Harter P., Marmé F., Jank P., Gaiser T., Neff C., Wagner U., Timms K.M., Rodepeter F. (2022). Homologous recombination deficiency as an ovarian cancer biomarker in a real-world cohort: validation of decentralized genomic profiling. J. Mol. Diagn..

[b0035] González-Martín A., Pothuri B., Vergote I., DePont C.R., Graybill W., Mirza M.R., McCormick C., Lorusso D., Hoskins P., Freyer G., Baumann K., Jardon K., Redondo A., Moore R.G., Vulsteke C., O'Cearbhaill R.E., Lund B., Backes F., Barretina-Ginesta P., Haggerty A.F., Rubio-Pérez M.J., Shahin M.S., Mangili G., Bradley W.H., Bruchim I., Sun K., Malinowska I.A., Li Y., Gupta D., Monk B.J. (2019). PRIMA/ENGOT-OV26/GOG-3012 investigators. Niraparib in patients with newly diagnosed advanced ovarian cancer. N. Engl. J. Med..

[b0045] Moore K., Colombo N., Scambia G., Kim B.G., Oaknin A., Friedlander M., Lisyanskaya A., Floquet A., Leary A., Sonke G.S., Gourley C., Banerjee S., Oza A., González-Martín A., Aghajanian C., Bradley W., Mathews C., Liu J., Lowe E.S., Bloomfield R., DiSilvestro P. (2018). Maintenance Olaparib in patients with newly diagnosed advanced ovarian cancer. N. Engl. J. Med..

[b0055] Ray-Coquard I., Pautier P., Pignata S., Pérol D., González-Martín A., Berger R., Fujiwara K., Vergote I., Colombo N., Mäenpää J., Selle F. (2019). Olaparib plus bevacizumab as first-line maintenance in ovarian cancer. N. Engl. J. Med..

[b0060] Witjes V.M., van Bommel M.H.D., Ligtenberg M.J.L., Vos J.R., Mourits M.J.E., Ausems M.G.E.M., de Hullu J.A., Bosse T., Hoogerbrugge N. (2022). Probability of detecting germline BRCA1/2 pathogenic variants in histological subtypes of ovarian carcinoma. A meta-analysis. Gynecol. Oncol..

